# Chemometric Analysis of UV-Visible Spectral Fingerprints for the Discrimination and Quantification of Clinical Anthracycline Drug Preparation Used in Oncology

**DOI:** 10.1155/2021/5580102

**Published:** 2021-05-06

**Authors:** Aimen El Orche, Casimir Adade Adade, Hafid Mefetah, Amine Cheikh, Khalid Karrouchi, Miloud El Karbane, Mustapha Bouatia

**Affiliations:** ^1^Laboratory of Chemical Processes and Applied Materials, Faculty of Science and Technology, Sultan Moulay Slimane University, Beni-Mellal, Morocco; ^2^Team of Formulation and Quality Control of Health Products, Faculty of Medicine and Pharmacy, Mohammed V University in Rabat, Morocco; ^3^Rabat Pediatrics Hospital, Ibn Sina University Hospital Center, Rabat, Morocco; ^4^Departement of Pharmacy, Faculty of Pharmacy, Abulcasis University, Rabat, Morocco

## Abstract

In clinical treatment, the analytical quality assessment of the delivery of chemotherapeutic preparations is required to guarantee the patient's safety regarding the dose and most importantly the appropriate anticancer drug. On its own, the development of rapid analytical methods allowing both qualitative and quantitative control of the formulation of prepared solutions could significantly enhance the hospital's workflow, reducing costs, and potentially providing optimal patient care. UV-visible spectroscopy is a nondestructive, fast, and economical technique for molecular characterization of samples. A discrimination and quantification study of three chemotherapeutic drugs doxorubicin, daunorubicin, and epirubicin was conducted, using clinically relevant concentration ranges prepared in 0.9% NaCl solutions. The application of the partial least square discriminant analysis PLS-DA method on the UV-visible spectral data shows a perfect discrimination of the three drugs with a sensitivity and specificity of 100%. The use of partial least square regression PLS shows high quantification performance of these molecules in solution represented by the low value of root mean square error of calibration (RMSEC) and root mean square error of cross validation (RMSCECV) on the one hand and the high value of *R*-square on the other hand. This study demonstrated the viability of UV-visible fingerprinting (routine approach) coupled with chemometric tools for the classification and quantification of chemotherapeutic drugs during clinical preparation.

## 1. Introduction

Currently, oncology treatment studies have become very important. Unfortunately, the number and cost of new anticancer drugs are still higher than ever before. This constitutes an economic challenge for countries with limited resources [1].

Daunorubicin, doxorubicin, and epirubicin belong to the family of anthracyclines used primarily as first-line drugs in combination with other chemotherapeutic drugs for a wide range of cancers, including breast cancer, bladder cancer, soft tissue and bone sarcomas, malignant lymphomas, and acute lymphocytic leukemia [2]. However, these therapeutic compounds have serious side effects: cardiotoxicity and myelosuppression are dependent on the dose [3]. These three anthracycline therapeutic molecules have similar molecular properties, which pose a great challenge for their discrimination.

The preparation of these anticancer drugs represents an important step in oncological treatment. Generally, this preparation has to comply with several rules and objectives; the safety of all personnel involved in terms of exposure, patient safety, and good production practices [4]. This preparation work must be carried out in a well-controlled atmosphere, with standardization of practices and the establishment of an effective system for monitoring and checking preparation quality [5, 6]. However, there remains a residual proportion of major or minor errors leading to nonconformity, despite all the precautions taken in a centralized unit.

In order to guarantee the pharmaceutical and chemical quality of the preparation, several strategies of quality control have been elaborated to make sure that the right drug is delivered at the right concentration [7].High-performance liquid chromatography coupled with UV (HPLC/UV) has been developed for proper drug preparation in chemotherapy and is considered one of the earliest methods used for monitoring such drugs [8–11]. In addition, other spectroscopic methods, such as UV-visible, near infrared, and Raman [12–15], have also been applied for the quality control of drug preparation in chemotherapy.

High-performance liquid chromatography coupled with spectrometry is a well-established approach for monitoring hospital chemotherapy preparations [16]. Generally, these analytical methods based on chromatography require the use of reagents which are sometimes expensive and harmful to the environment, time consuming to carry out an analytical test, and require qualified operators. For this reason, the development of fast and environmentally friendly methods constitutes a great challenge in the field of analytical chemistry. Recently, new technology has been emerged to satisfy this demand, and these methods are based on the combination of nondestructive spectroscopic methods with the recognition algorithms such as PLS-DA, PLS, and support vector machine for the classification between groups of individuals and the quantification of their content in a given matrix according to spectral data [17–19].

Today, UV-visible spectroscopy combined with chemometric classification algorithms is widely used to develop rapid methods for drug quality control [20] and well established in the pharmaceutical industry to identify raw materials prior to production, falsified drugs, and also in process analytical technology.

This study focused on three chemotherapeutic drugs that are very similar in physicochemical properties and are difficult to discriminate: epirubicin, doxorubicin, and daunorubicin. These drugs are anthracyclines and antitumor antibiotics consisting of a planar anthraquinone ring attached to a carbohydrate containing an amino group. Doxorubicin is a natural product obtained from Streptomyces galilaeus while epirubicin is its semisynthetic analogue which differs only in its stereochemistry. These molecules function by intercalating DNA strands and leading to the formation of complexes that inhibit DNA and RNA synthesis. Daunorubicin is an anticancer drug used in the treatment of acute myeloblastic leukemia and acute lymphoblastic leukemia. Daunorubicin (DNR) derived from the fermentation of Streptomyces strains and was the first anthracycline used in clinical practice [15, 21]. It is an agent that causes the blockage of DNA replication and the accelerated cell death of cancer cells. This drug is very often formulated in our hospital for the treatment of cancer diseases.

The aim of this study was to confirm the ability of UV-visible spectroscopy combined with multivariate data analysis such as PCA, PLS-DA, and PLS to monitor three anthracycline drugs at different concentrations, in order to examine the ability of this technology to discriminate and quantify these three drugs, which are considered among the most crucial to distinguish between them due to their similar chemical structures and their low concentrations.

## 2. Materials and Methods

**Figure 1:**
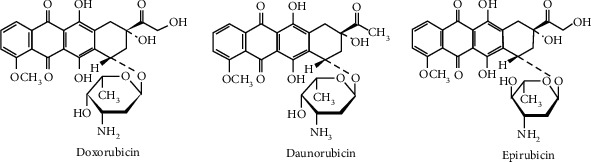
Molecular structure representation of the three drugs of the anthracycline family.

Figure 1 shows an illustration of the molecular structure of daunorubicin, doxorubicin, and epirubicin that belongs to the anthracycline family used in chemotherapy. These three molecules are used to treat various types of cancer. These drugs have a similar molecular structure, as well as common physicochemical properties, which makes their discrimination difficult in the clinical chemotherapy preparation.

### 2.1. Sample Preparation

Several commonly used anthracyclines (Ddunorubicin, doxorubicin, and epirubicin) have been diluted at different concentrations with 0.9% sodium chloride which are usually used for this type of reconstitutions. Subsequently, they were analyzed directly using a double-beam spectrophotometer UV-visible Perkin-Elmer Lambda 12, at a wavelength ranging from 350 to 800 nm.

The study was performed at different concentrations corresponding to (0.001-0.0064 mg/mL for doxorubicin, 0.006-0.077 mg/mL for daunorubicin and 0.006-0.083 mg/mL for epirubicin). A calibration model was developed by PLS-DA, and then crossvalidation was used to validate these models. For the construction of the PLS-DA and PLS regression model, a database of 52 samples has been used (22 of doxorubicin, 15 of daunorubicin, and 15 of epirubicin).

### 2.2. Multivariate Data Analysis

Chemometrics, also known as multivariate data analysis, is the science that implements optimal mathematical and statistical approaches to process chemical data. Chemometrics involves experiment design upstream and data processing to obtain valuable information once measurements have been taken. The growing need for chemometric tools comes especially through the development of analytical equipment that provides big amounts of complex data [22].

This scientific field comprises a wide variety of statistical methods, aimed at analyzing numerous data sets especially spectral data such as infrared spectroscopy, fluorescence, and UV-visible spectroscopy to achieve several objectives.

### 2.3. Partial Least Squares Regression

Partial least squares (PLS) regression is a chemometric technique often used for data modeling. PLS allows the reduction of predictors to a smaller set of noncorrelated components called latent variables (LVs) and performs the least squares regression on these main principal components (LV). The PLS feature is especially useful when the initial variables (predictors) are strongly correlated, or when the number of predictors exceeds the number of observations. PLS regression can be summarized in two related steps:

Dimensionality reduction of the *X* (initial data matrix) and *Y* (response matrix) matrix into latent variables using score projections. Then, the incorporation of a vector, known as weight (w), relates to the two latent variable matrices and providing the covariance between the *X* and *Y* scores as an optimization criterion.

### 2.4. Partial Least Squares Discriminant Analysis

The partial least squares (PLS) algorithm was initially implemented for the regression task and subsequently evolved into a well-known classification method known as PLS discriminant analysis (PLS-DA). PLS-DA integrates dimensionality reduction and discriminant analysis into a unique algorithm and is especially applicable to high-dimensional data modeling [23].

Traditionally, PLS has been proposed to deal with continuous variables (i.e., a stain) in regression. But in the cluster task, the output variables will be always categorical. Hence, the first step in PLS-DA modeling is to recode the categorical variables (i.e., ordinal or nominal) into continuous (i.e., numerical) variables [23, 24].

Two principal algorithms can be applied to perform PLS regression: an iterative procedure is known as the NIPALS algorithm (nonlinear iterative partial least squares) [25], or a direct method, known as the SIMPLS algorithm [26]. In this study, the PLS regression was performed using the NIPALS algorithm.

### 2.5. Software

All data analysis of UV-visible spectra and chemometric applications, PCA, PLS-DA, and PLS regression, were performed using Unscrambler software, version 10.4 camo analytic.

## 3. Results and Discussion

**Figure 2:**
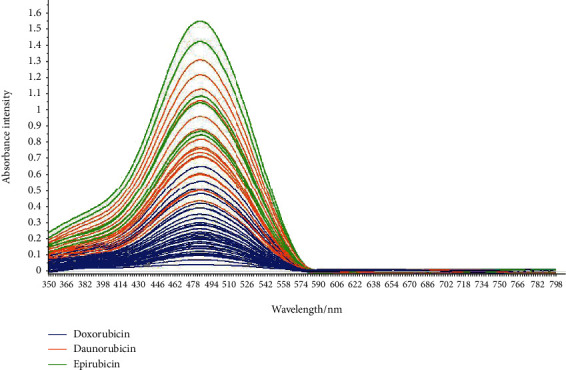
Graphical representation of the UV-visible absorption spectra of the three molecules.

According to the UV-visible spectrum of the molecule, we notice that these three molecules absorb in a similar wavelength region with maximum absorption in 480 nm as shown in Figure 2, and this is explained by the high number of chromophores present in the three drugs (Figure 1). However, observable differences have been noticed in the spectral intensity, but this difference is due to the difference between the preparation concentrations of these molecules, which are prepared in different concentrations.

Due to the similarity shown in the molecular structure of the three molecules belonging to the anthracycline family, it is very difficult to discriminate between these three molecules during the pharmaceutical preparation using UV-Visible spectroscopy.

### 3.1. Principal Component Analysis (PCA)

In Figure 2, the resulting UV-Visible spectra show no significant differences between the three drugs, and only differences in absorption intensity were visually observable. The observation of spectral differences between the samples was difficult to see with the naked eye when these drugs are prepared in close or similar concentrations, and for this reason, chemometric statistical methods can be used to help observe the variability of the spectral data. Chemometric statistical methods include PCA. This method can be used to cluster samples and observe clustering relationships between samples based on differences in the UV-visible spectral data of each sample. The PCA method was mainly used to reduce the dimensionality of data in order to obtain a better visualization about individual's similarity, group's classification, and variable correlation.

**Figure 3:**
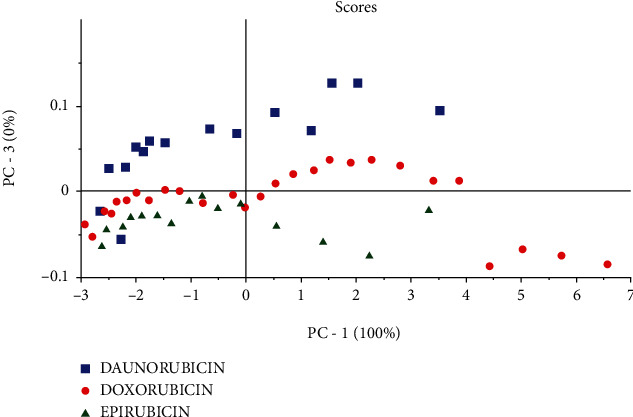
PCA score plot results of two principal components (PC1-PC3).

PCA analysis revealed that the variability distribution was mainly given by the first principal component PC1, which represents 100% of total variability. The use of the score plot (Figure 3) demonstrates that there is a classification between the three drugs, and this classification was generally considered very weak, due to the similar physicochemical properties of the drugs.

PCA analysis shows that the first component gives useful information about the concentration of these drugs, and samples of high concentration contribute substantially and positively with the PC1 axis while samples of low concentration contribute negatively with the PC1 axis.

### 3.2. Discriminant Analysis PLS-DA

In order to successfully develop classification models capable of determining the class of the three drugs, chemometric classification tools have been applied to the spectral data. In this study, PLS-DA was used to discriminate the three chemotherapeutic drugs. The correct classification rate % CCR, sensitivity, and specificity are the main parameters used to assess the discriminatory power of an analytical method.

**Figure 4:**
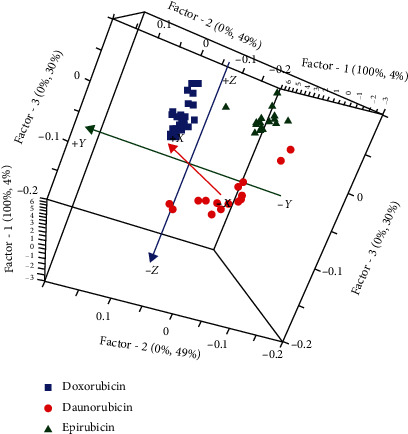
3D-PLS-DA score plot for the UV-visible spectra of the three chemotherapy drugs.

The observation of the 3D score plot generated by PLS-DA (Figure 4) shows a perfect discrimination between the three groups of chemoterapic drugs. The evaluation of the quality of the constructed model was demonstrated by the high value of *R*-square and the low value of root mean square of calibration RMSEC and crossvalidation RMSECV.

Assessment of the performed models reveals that the correlation coefficient is between 99% and 98% for the calibration results and between 98% and 96% for the crossvalidation results, whereas the root mean square error of the calibration is between 0.04 and 0.07 and for crossvalidation ranges between 0.06 and 0.08 (Table 1).

Eventually, the results were displayed in the form of confusion matrices, enabling the estimation of the correct classification rate, specificity, and sensitivity of the discrimination achieved across the test set.

According to the confusion matrix (Table 2), it can be seen that all individuals belong to their class with a CCR, specificity, and selectivity of 100%. The validation of this classification model by crossvalidation shows a high capacity in the classification of the three drugs of the anthracycline family with a classification accuracy that reaches 100%. Despite the similarities in structure between doxorubicin, epirubicin, and daunorubicin, since they belong to the same family of anthracyclines and anticancer drugs (56), the combination of UV-visible with PLS-DA was able to differentiate very clearly between these three drug formulations.

The combination of UV-visible spectral data with the PLS-DA method provides powerful results for the discrimination of the three drugs regarding specificity, sensitivity, and CCR. These results are generally consistent with other studies that have used factorial discriminant analysis (PCA-FDA) in combination with RAMAN and infrared spectroscopy [27]. Other studies also show a high capacity of the combination of PLS-DA with spectroscopic methods for the discrimination of drugs used in chemotherapy. Another study also shows a high capacity of the combination of PLS-DA with Raman spectroscopy for the discrimination of antineoplastic taxane drugs [15, 21].

### 3.3. Quantitative Analysis

In order to optimize the quantification models of the three drugs of the anthracycline family, a PLS regression method was applied to the UV-visible results. Numerous pretreatments were applied for the construction of the PLS models. The best quantification models were chosen on the basis of statistical parameters, and the best patterns are those having the lowest root mean square error of calibration (RMSEC), the lowest root mean square error of crossvalidation (RMSECV), and the highest *R*-square.

This table presents the performance parameters of the PLS quantification models and the number of latent variables (LVs) used to build the PLS models. It provides also information on the predictive performance results, including the coefficient of determination (*R*^2^) for calibration and crossvalidation and errors (RMSECV) (RMSEC).

**Figure 5:**
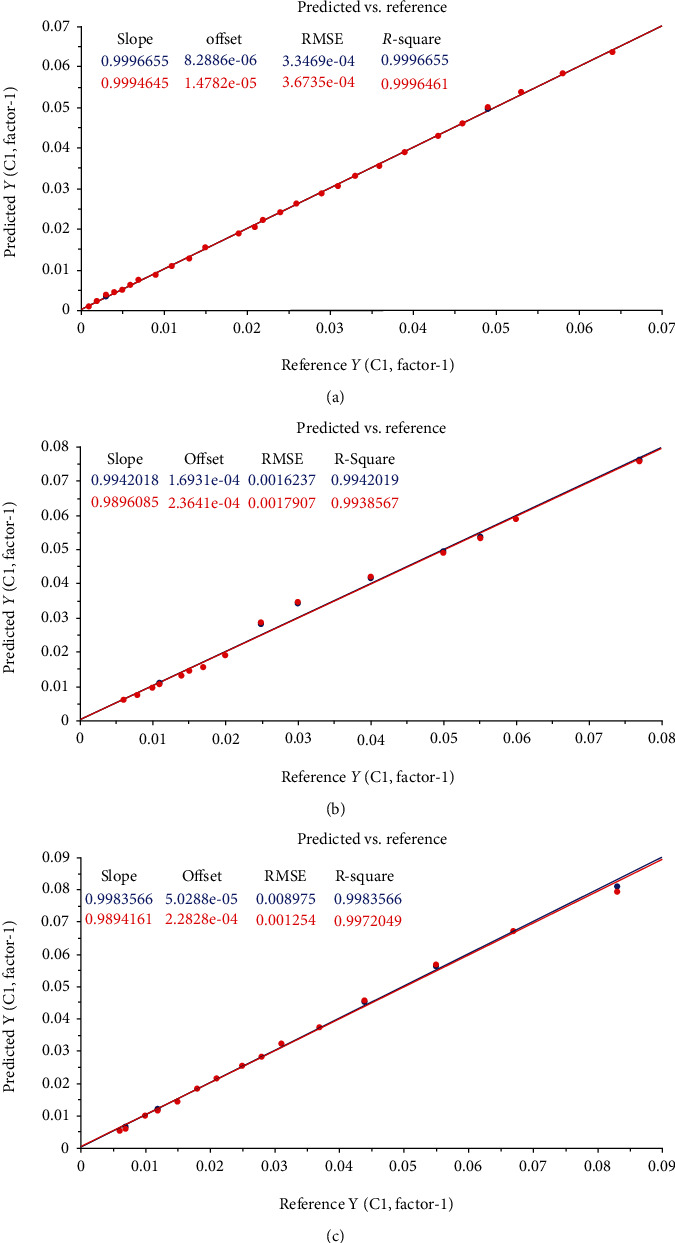
Actual values vs. predicted values using PLS regression ((a) doxorubicin, (b) daunorubicin, and (c) epirubicin).

The results found by PLS regression show high performance for the quantification of the three drugs using the first latent variable that represents 100% of the total variability of the data set. This performance is explained by the low value of RMSECV and RMSEC and the high value of *R*-square (Table 3). Therefore, the high value of *R*-square indicates strong linearity between the reference and predicted values as shown in Figure 5.

These results found are adequate with the results found by Makki et al., which shows an *R*-square of 0.99 and RMSECV ranging between 0.0127 and 0.0220 [27].

This study confirms the qualitative and quantitative capability of the combination of UV-visible spectroscopy and chemometric models to identify and verify cytotoxic drug preparations. Total discrimination was observed over the entire range of therapeutic concentrations, even for low concentrations, and no samples were misclassified. Strong linearity was observed for the quantification models over the entire concentration range. In light of these results, the use of UV-visible spectroscopy using chemometric methods is an excellent approach to ensure the nature of the drug prior to administration to patients.

Besides the analytical performance, the robustness of the instrument and its simplicity of use constitute favorable arguments for implementing the UV-visible portable scanner. With regard to the expected results, it will be valuable to expand this trial to additional drugs and containers used to deliver drug therapy in order to enhance safety management in the clinical drug preparation.

## 4. Conclusion

In this study, a rapid qualitative and quantitative approach has been developed for the discrimination and quantification of the content of three drugs used in oncology. The combination of UV-visible spectroscopy and chemometric methods shows high performance for the discrimination between the three drugs with a sensitivity and specificity of 100%.

The application of PLS regression also shows a high capacity in the quantification of these three drugs, and this capacity is explained by the low values of RMSECV and RMSEC as well as the high value of *R*-square.

As a general conclusion, the coupling of UV-visible spectra and suitable chemometric analysis tools, in particular PLS regression and PLS-DA, has demonstrated their efficiency for the discrimination and quantification of doxorubicin, daunorubicin, and epirubicin.

Similar multivariate models are strongly recommended as a clean, rapid, and reliable means of assessing the quality control of clinical chemotherapeutic preparations.

## Figures and Tables

**Table 1:** Statistical parameters of the PLS-DA model.

Label	Number of latent variable	Calibration		Crossvalidation	
		*R*-square	RMSEC	*R*-square	RMSECV
Doxorubicin	7 LV	0.99	0.05	0.97	0.08
Daunorubicin		0.98	0.07	0.96	0.09
Epirubicin		0.99	0.04	0.98	0.06

**Table 2:** Confusion matrix for the classification of anthracycline drugs (1: doxorubicin, 2: daunorubicin, and 3: epirubicin) using the PLS-DA method.

Confusion matrix	Actual set						
	Label	1	2	3	Specificity %	Sensitivity%	%CCR
Predicted training set	1	22	0	0	100	100	100
	2	0	15	0			
	3	0	1	15			

**Table 3:** Performance results of the quantification models developed for all concentration ranges of the three drugs.

	Doxorubicin	Daunorubicin	Epirubicin
Concentration range mg/mL	0.001-0.064	0.006-0.077	0.006-0.083
Spectral pretreatment	Raw	Raw	Raw
Number of variables latent	1	1	1
*R*-square of calibration	0.99	0.99	0.99
*R*-square of crossvalidation	0.99	0.99	0.99
RMSECV	0.00037	0.00179	0.00125
RMSEC	0.00033	0.00162	0.00089

## Data Availability

The data used to establish this research are available on request from the corresponding author.
